# Microenvironmental Control of High-Speed Interstitial T Cell Migration in the Lymph Node

**DOI:** 10.3389/fimmu.2016.00194

**Published:** 2016-05-13

**Authors:** Tomoya Katakai, Tatsuo Kinashi

**Affiliations:** ^1^Department of Immunology, Graduate School of Medical and Dental Sciences, Niigata University, Niigata, Japan; ^2^Department of Molecular Genetics, Institute of Biomedical Science, Kansai Medical University, Hirakata, Japan

**Keywords:** adhesion, chemokine, dendritic cell, fibroblastic reticular cell, integrin, migration, lymph node, T cell

## Abstract

T cells are highly concentrated in the lymph node (LN) paracortex, which serves an important role in triggering adoptive immune responses. Live imaging using two-photon laser scanning microscopy revealed vigorous and non-directional T cell migration within this area at average velocity of more than 10 μm/min. Active interstitial T cell movement is considered to be crucial for scanning large numbers of dendritic cells (DCs) to find rare cognate antigens. However, the mechanism by which T cells achieve such high-speed movement in a densely packed, dynamic tissue environment is not fully understood. Several new findings suggest that fibroblastic reticular cells (FRCs) and DCs control T cell movement in a multilateral manner. Chemokines and lysophosphatidic acid produced by FRCs cooperatively promote the migration, while DCs facilitate LFA-1-dependent motility *via* expression of ICAM-1. Furthermore, the highly dense and confined microenvironment likely plays a key role in anchorage-independent motility. We propose that T cells dynamically switch between two motility modes; anchorage-dependent and -independent manners. Unique tissue microenvironment and characteristic migration modality of T cells cooperatively generate high-speed interstitial movement in the LN.

## Introduction

In addition to their strategic locations throughout the lymphatic vascular system, lymph nodes (LN) contain a variety of immune cells, chiefly lymphocytes, which make them an ideal device for coupling lymph fluid filtration to the collection of antigens and induction of adaptive immune responses (1, 2). Dendritic cells (DCs) that have captured antigens in peripheral tissues migrate to LN through lymphatic vessels and enter the paracortex, a T cell rich area. In the paracortex, antigen-presenting DCs initiate adaptive immune responses by activating T cells (1–4). The likelihood of T cells encountering cognate antigens in the LNs is dramatically increased because antigen-presenting cells and T cells are highly concentrated within the restricted area. However, it alone is probably insufficient for the detection of antigens by rare antigen-specific T cells efficiently.

Since 2002, it became common to perform live imaging using a two-photon laser scanning microscope to observe surgically exposed LN in an anesthetized mouse (intravital) or explanted LN under perfusion (5–7). These observations revealed robust migration of lymphocytes within LN, often called interstitial or intranodal migration. In particular, T cell movements in the paracortex occurred in a non-directional manner at high velocities (average >10 μm/min), and T cells made contact with large numbers of DCs over a limited period of time (5, 8–10). Given the extremely low frequency of naive T cells capable of recognizing each antigen (11–13), active migration by T cells is likely essential for the efficient detection of rare cognate antigens. However, the mechanism that enables T cells to achieve high-speed movements within a densely packed tissue environment is not fully understood. Considering intranodal T cell migration (INTM) in the context of tissue microenvironment is beneficial to know about it. In this perspective article, we will discuss the various factors in the microenvironment of LN paracortex that control the efficient movement of T cells.

## Migration of Lymphocytes *in vitro*

Based on examinations in two-dimensional (2D) environments *in vitro*, it became evident that immune cells show higher motility than many other tissue cells (14, 15). Various stimuli increased leukocyte motility and changed their morphology to an elongated shape with clear front–rear asymmetry. Changes in morphology depend on the remodeling of actin cytoskeleton and cycles of elongation–contraction motion mediated by actomyosin machinery (14, 16). Therefore, high motility is likely due to a high turnover rate or remodeling of the cytoskeletal machineries. In addition, relatively weak adhesiveness in hematopoietic cells could be a prerequisite for raising migration velocity (15, 17).

Chemokines are key regulators of immune cell trafficking and tissue localization (18). Chemokine receptors transduce migratory stimuli and initiate signaling cascades that culminate in cytoskeletal remodeling and morphological changes (14, 16). In lymphocytes, Gαi-coupled chemokine receptors are connected to a guanine–nucleotide exchange factor DOCK2 and Rac small GTPases, which promote actin dynamics to form lamellipodia in the cell front (19–21). In the rear of the cell, the activation of Rho small GTPases and non-muscle myosin II (nmMyoII) drive the elongation–contraction cycle (14, 16, 21). Front–rear polarity and integrin-dependent adhesion are also regulated by the small GTPase Rap1 (22–24).

**Figure 1:**
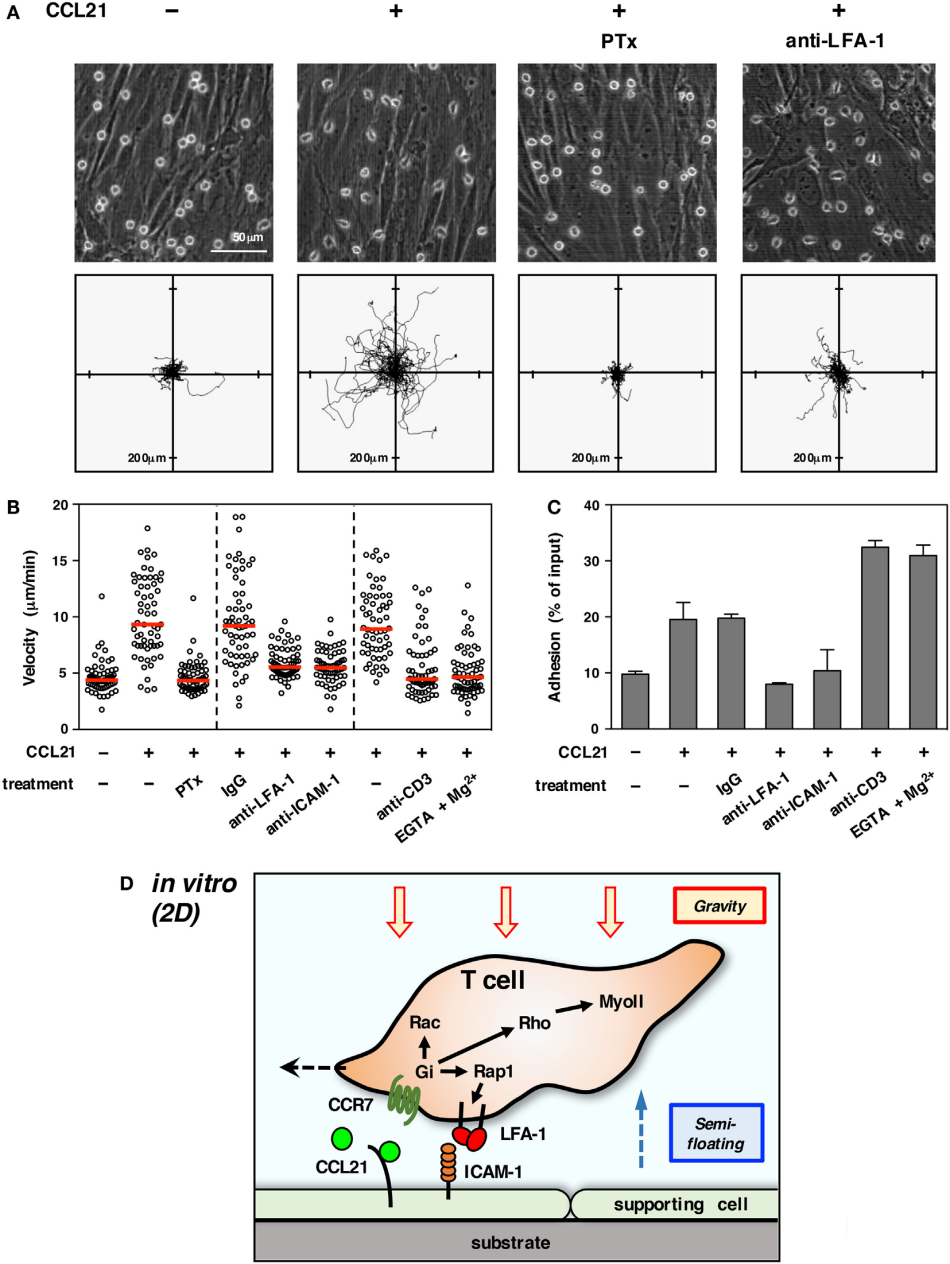
**T cell migration *in vitro* (2D environment)**. **(A)** Chemokine-induced morphology and motility of T cells on primary LN stromal cells. To form a primary LN stromal cell monolayer, CD45^−^ cells were isolated from C57BL/6 mice LNs by magnetic cell sorting and cultured on chamber dishes for 7–10 days (27). Total T cells were isolated from LNs by magnetic cell sorting and applied onto the stromal monolayer in the presence or absence of CCL21 (100 nM). Cell motility was examined using time-lapse video recording at 37°C (28). Representative static views (upper panels) and cell trajectories (lower panels) are shown (*n* ≧ 3). In some cases, T cells were pretreated with pertussis toxin (PTx) to inhibit Gαi or anti-LFA-1 antibody was added to medium to block LFA-1 function. Note that CCL21 induces a characteristic polarized morphology and stimulates the motility of T cells. Gαi inhibition completely abrogates both morphological changes and migration. LFA-1 blockage dramatically inhibits migration but not polarization, indicating that adhesion mediated by LFA-1 to stromal ICAM-1 is required for T cell movement in this setting. **(B)** Various treatments affect T cell migration on a LN stromal monolayer [partially adapted and modified from Ref. (27)]. The mean velocity of individual cells (circles) and the median (horizontal red bars). Representative results of more than three experiments are shown. Note that the inhibition of Gαi completely blocks T cell migration. Antibody blockade of LFA-1 or ICAM-1 dramatically reduces motility. The treatment of T cells with anti-CD3 antibody or EGTA + Mg^2+^, both of which induce high-affinity integrin activation and strong adhesion, inhibits T cell migration. **(C)** Adhesion of T cells to a stromal monolayer in various settings. Fluorescent-labeled T cells were applied onto a LN stromal monolayer and incubated in the presence or absence of the indicated treatments for 3 h (28). Non-adherent T cells were washed three times, and the fluorescence of remaining cells was measured to reflect the intensity of T cell adhesiveness. Results are shown as mean ± SD. Note that anti-CD3 antibody or EGTA + Mg^2+^ both induced strong T cell adhesion, while CCL21 alone stimulated relatively weak adhesiveness in a LFA-1/ICAM-1-dependent manner. **(D)** Schematic representation of T cell migration in a 2D environment. Due to gravitational force, T cells are settled on the surface of a supporting cell monolayer in the bottom of the cell culture chamber. Intracellular signals transduced from Gi-coupled chemokine receptors induce actin reorganization, front–rear asymmetry, actomyosin contraction, and LFA-1-dependent dynamic adhesion to ICAM-1 on stromal cells *via* activation of small GTPases. The high LFA-1 dependency in 2D migration assays is likely due to the requirement of anchorage to supporting cells for forward movement under semi-floating condition in culture media.

Importantly, lymphocyte migration in 2D conditions *in vitro* revealed a marked dependency on adhesion to supporting cells (15, 25) (Figure 1). In particular, the integrin LFA-1 (αLβ2) plays a pivotal role in the motility of T cells on some supporting cells or immobilized ligands (16, 22, 23, 26). Extracellular stimuli, such as chemokines, are shown to facilitate LFA-1-dependent adhesiveness to ICAM-1. Thus, the first issue needed to be addressed by live imaging was to determine whether T cells moving in LN employed the same molecular mechanisms as *in vitro*.

## Mechanism of Interstitial T Cell Migration in LN

Based on two-photon imaging, it has been revealed that INTM involves intracellular regulators that play a role in associated with actin reorganization. For example, the migration of DOCK2- or Rac1/Rac2-deficient T cells was severely impaired (29, 30). Likewise, T cells deficient in an actin regulator coronin 1A displayed a severe impairment in intranodal motility (31). The requirement of PI3K, a classical regulator of actin, in INTM was also suggested, although there are some conflicting reports (29, 32, 33). Moreover, Mst1, an effector kinase downstream of Rap1, which is a critical regulator of integrin and polarity formation, is necessary for optimal INTM (34).

Chemokine signaling controls actomyosin machinery that mediates both directional movements along a gradient (chemotaxis) and non-directional migration under uniform concentrations (chemokinesis) that is reminiscent of T cell migration in LNs. In fact, the inhibition of Gαi-coupled receptors by pertussis toxin markedly reduced INTM by 40–50% in velocity (35, 36). Similarly, reduced motility was also observed in Gαi2^−/−^ T cells (37). In the paracortex, stromal cells called fibroblastic reticular cells (FRCs) produce the chemokines CCL19 and CCL21, which control the localization of T cells expressing the cognate receptor CCR7 (38–40). Wild-type T cells in LN from CCL19/CCL21-deficient mice or CCR7^−/−^ T cells in wild-type LNs showed 20–35% migration reduction, indicating that CCR7 ligands play a role in INTM to some extent (35, 36, 41).

Given that chemokines induced LFA-1-dependent adhesion (16, 42), LFA-1 was also expected to participate in INTM. However, T cells from LFA-1-deficient (β2^−/−^) mice that were transferred to wild-type mice showed only slight reductions in the velocity of INTM by 15% (43). In the same report, the authors suggested that integrins are “silent” on chemokine-stimulated T cells under share-free condition, based on the observation that immobilized chemokines were able to induce motility but not firm adhesion *in vitro*, and most T cells seemed to keep migrating without any arrest in the LN, in which CCL21 is immobilized on FRCs. Furthermore, another group demonstrated that DCs lacking all functional integrins exhibited ability to migrate into and within LN (44). Consequently, these findings broadened the understanding that integrins are dispensable for interstitial motility of leukocytes.

## Adhesion-Independent Motility in Confined Environment

Elaborate networks constructed by FRCs and extracellular matrix backbone support the tissue framework of the LN paracortex, whereas most of the matrix fibers (collagen, laminin, fibronectin, etc.) are enclosed by FRCs, preventing T cells from direct contact to the fibers (45–48). The facts that FRCs produced chemokines and integrin ligands (38, 46) and most T cells appeared to migrate along the network (48) led to the notion that T cells might adhere to FRC surface as foothold. Seemingly, random migration was supposed as a guided movement on highly branched network. However, as the requirement of integrins became questionable, arguments regarding the mechanism of INTM have changed directions. It became evident that a variety of cells often showed anchorage-independent motility in some three-dimensional (3D) confined environments enclosed by matrices or artificial substrates (49, 50). Likewise, lymphocytes exhibited significant motility in confined environments without remarkable adhesiveness (14, 51). In a narrow microchannel enclosed by walls of resin with little adhesiveness, lymphocyte increased motility in a range of space around 1-cell diameter and reduced as the space was expanded (52, 53). Consequently, INTM became considered an anchorage-independent motility in a kind of confined environment. However, individual T cells present in the LN parenchyma are surrounded by cells with significant plasticity and dynamics (48), which possibly makes confinement irregular or unstable.

## Reconsideration of LFA-1/ICAM-1 Axis in Interstitial T Cell Migration

**Figure 2:**
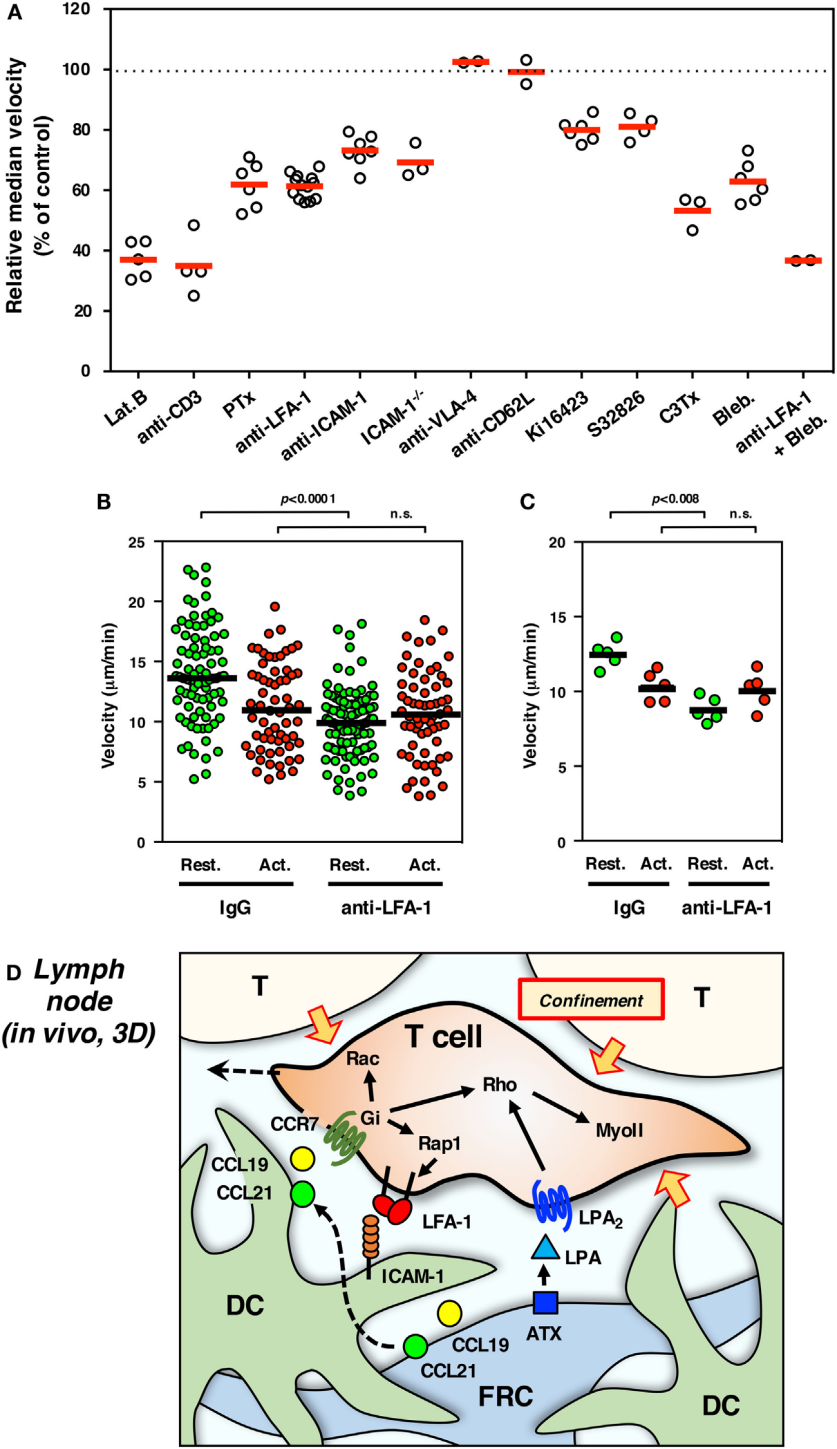
**Interstitial T cell migration in LN**. **(A)** Various treatments affect T cell migration in LN slices [partially adapted and modified from Ref. (27, 54)]. The circles represent the migration velocity (%) compared to the control in each experiment, and the horizontal red bars represent the mean (*n* ≧ 2). Lat.B, latrunclin B (actin inhibitor); Ki16425, LPAR inhibitor; S32826, ATX inhibitor; C3Tx, C3 toxin (Rho inhibitor); Bleb., blebbistatin (nmMyoII inhibitor). **(B,C)** Migration velocities of resting (Rest., green) and activated (Act., red) T cells in LN slices in the presence of control IgG or anti-LFA-1 antibody. The plot in **(B)** shows the mean velocity of individual cells (circles) and the median (horizontal bars), while the plot in **(C)** shows the median velocity for individual experiments (circles) and the mean of five experiments (*n* = 5, horizontal bars). For activation, total T cells isolated from LNs were stimulated with immobilized anti-CD3 and soluble anti-CD28 antibodies for 3 days. Freshly isolated resting T cells and activated T cells were labeled with different fluorescent dyes (CFSE and CMTMR), mixed at equal numbers, and applied to LN slices for the examination by two-photon laser scanning microscopy. The trajectory data sets of resting and activated T cells in each treatment were obtained from the same image field. Note that resting T cells but not activated T cells show the reduction of velocity in response to LFA-1 blockade, suggesting a distinct difference for LFA-1-dependent “speed-up” in resting but not activated T cells. Statistical analysis: Mann–Whitney *U* test. n.s., not significant. **(D)** Schematic representation of microenvironmental cues for high-speed interstitial T cell migration in the LN paracortex. FRCs produce chemokines (CCL19 and CCL21) and ATX (LPA), while DCs produce CCL19 but bind FRC-derived CCL21 on surface glycans. The CCR7 ligands input migratory signals in T cell, which induce actin reorganization, front–rear asymmetry, actomyosin contraction, and LFA-1-dependent dynamic adhesion to ICAM-1 on DC through the function of small GTPases. LPA plays additive or compensatory role to stimulate actomyosin-mediated motility and cellular deformation to adopt the complicated geometry and confinement of tissue microenvironment.

Strong adhesiveness naturally hinders or stops T cell migration, whereas weak and transient anchorage to substrate is in turn likely to generate a traction force for forward movement even in a 3D environment. From this viewpoint, LFA-1 is quite suitable for anchorage-dependent rapid movement, because of their unique property to quickly respond to external stimuli (16, 23). In order to evaluate the significance of LFA-1 in INTM, we constructed an imaging system using LN slices (54). The upper part of the LN was removed to expose the tissue parenchyma, and T cells were added directly to tissue. Using this model, the inherent biases of LN homing across high endothelial venules were alleviated. T cells were applied to the LN slices in the absence or presence of antibodies or drugs for the rapid inhibition of migration machineries. In this system, LFA-1 inhibition with antibody constantly reduced migration by 30–40% (Figure 2A) – in particular the high-speed fraction (>10 μm/min) and relatively straight movement were clearly decreased (54). Motility of wild-type T cells was similarly reduced in ICAM-1^−/−^ LN slices. These suggest that LFA-1/ICAM-1-mediated adhesion plays a significant role in INTM, at least in this setting.

What brings the difference between the previous works and results in LN slice? According to Woolf et al., β2^−/−^ T cells also showed a slight but significant reduction of migration velocity, suggesting that LFA-1 promoted migration (43). On the other hand, β2^−/−^ mice exhibited a severe disturbance in the immune system (55), which raises concerns regarding the nature of lymphocytes. In β2^−/−^ mice, T cells develop and are able to distribute to LNs, despite inefficiency in the homing step that normally requires LFA-1 (16, 23). It is important to note that β2^−/−^ T cells that enter wild-type LNs in the absence of LFA-1 may be enriched with a relatively high motility population that leads to the underestimation of LFA-1 function in INTM. Moreover, besides the strong binding responsible for cell arrest, LFA-1 also mediates dynamic adhesion mode characterized by relatively weak binding, which is reflected in rapid lymphocyte migration with remarkable LFA-1 dependency *in vitro*. Therefore, migration without arrest does not necessarily indicate the silence of LFA-1. Finally, even if DC motility does not require integrins, it is still questionable whether the mechanism is applicable to INTM as the speed and migrating morphology of DCs markedly differ from that of T cells (44).

On the other hand, there are a number of limitations associated with the use of LN slices. There is a possibility that cutting the organ causes some physical and physiological changes that affect T cell migration; for example, reduced internal pressure could expand the space between cells, which might increase LFA-1 dependency. However, this is not likely because similar results were obtained by conventional methods using LN explants or intravital observations (54). Antibodies or chemical inhibitors that are applied to LN slices could affect whole tissues as well as T cells of interest, which may in turn impact cell pressure generated by “push and shove” from surrounding cell mass. In fact, even if voluntary movement is virtually blocked, the cell still shows a residual mobility or vibrating motion; thereby, velocity does not drop to 0 (Figure 2A). Non-voluntary movement in INTM is quite important because it could be 1/4 of motility represented by velocity. Nevertheless, considering that some reports also demonstrated the contribution of ICAM-1 in INTM (56–58), LFA-1 dependency might be variable depending on experimental settings. In any cases, the contribution of LFA-1/ICAM-1 system is clearly partial ranging between 10 and 40% in INTM, which differs from 2D situation *in vitro* (Figure 1).

For the imaging of INTM, T cells that are used for transfer to mice often contain a mixed population that is composed of naive CD4^+^ and CD8^+^ T cells and activated/memory T cells. It is worth noting that LFA-1-dependent fraction of motility is completely disappeared in activated T cells (Figures 2B,C). This finding raises a possibility that differences in the proportion of T cell subsets and their activation status could give rise to altered results in INTM.

## ICAM-1 on DCs Mediates LFA-1-Dependent Motility

Given that LFA-1 participates in INTM, FRCs with high expression of ICAM-1 were considered target footholds for LFA-1-mediated anchorage. However, many DCs localized to the same area with much higher ICAM-1 expression. To determine which is crucial for LFA-1-dependent motility, bone marrow chimeras using wild-type and ICAM-1^−/−^ mice were made for analysis (27). Wild-type T cells in the LN slices from wild-type mice reconstituted with ICAM-1^−/−^ bone marrow showed reduced motility, whereas T cells in the LNs of ICAM-1^−/−^ mice reconstituted with wild-type bone marrow did not demonstrate reduced motility. This indicates that ICAM-1 expressed by hematopoietic cells, but not by radioresistant cells, including FRCs is important. Moreover, in an ICAM-1^−/−^ environment, T cells restored migration when ICAM-1 was expressed by DCs. Therefore, ICAM-1 displayed on DCs supports LFA-1-dependent T cell motility in the LN paracortex. The total surface area of DCs is estimated to be larger than FRCs and most part of FRC network is covered with DCs, suggesting that T cells are likely to contact with DCs more frequently than FRCs. DCs are CCL19 producers, while they do not express CCL21 but instead bind it onto surface glycans (4, 59). Together, it is reasonable to assume that by touching DCs, T cells receive chemokine signals that in turn stimulate dynamic anchorage *via* LFA-1 to grip on ICAM-1 and move forward over the DCs.

## Motility Independent of Chemokine Signal or LFA-1

Even though chemokine signaling and LFA-1/ICAM-1 are inhibited, T cells still retain substantial motility. We speculated that this residual motility was due to some unknown factor(s) produced by FRCs. Microarray analysis of LN FRCs revealed high expression of autotaxin (ATX) (27). ATX is an ectoenzyme that generates lysophosphatidic acid (LPA), a lipid mediator known to promote motility in various cells including T cells (60–62). Pharmacological inhibitors against ATX or LPA receptors decreased INTM in LN slices by ~20% (Figure 2A), and acted in concert with pertussis toxin or LFA-1 blockade to further decreased T cell motility (27). Therefore, it is likely that ATX/LPA signaling plays a role in Gαi- or LFA-1-independent motility. LPA was shown to promote T cell chemokinesis and LPA_2_ was recently identified as LPA receptor responsible for optimal INTM (27, 62–64).

LPA as well as CCL21 activate Rho in T cells, and the simultaneous stimulation results in an additive effect on Rho activation (27, 65, 66). Rho function is crucial for INTM because the inhibition of Rho by C3 toxin markedly reduces interstitial motility through the suppression of rear contractility. Pharmacological inhibition of nmMyoII also inhibits INTM and further decreases motility with LFA-1 blockade (27) (Figure 2A). Therefore, the Rho-nmMyoII pathway is indispensable for efficient INTM, especially in the LFA-1-independent fraction of motility that is mediated in part by ATX/LPA signaling.

## Migration Modalities in Specialized Tissue Microenvironment

Actomyosin machinery is a crucial component in anchorage-independent motility in spatially restricted environments that require amoeboid or squeezing deformation of cells (25, 44). This type of migration modality is probably required for T cells moving through, as avoiding obstacles, in the LN parenchyma, in which numerous swarming lymphocytes and supporting cell networks are densely packed into a complicated environment (52, 64). In general, Rho- and nmMyoII-mediated rear contraction in migrating cells is considered to be required for removing adhesion from a substrate (58, 67–69), but it is unclear how effective it is in rapidly moving naive T cells with weak adhesiveness in interstitium. Meanwhile, actively migrating T cells in a confined environment exhibit a “walking” like behavior by touching substrates intermittently with small parts of the cell (52, 53, 69). A wavy surface and contraction of cell body generated by actomyosin function also facilitate a characteristic wiggling or squirming motions (52, 70). Thus, actomyosin-driven continuous deformation and frequent changes in direction are probably important for efficient movement by reducing excess adhesiveness and avoiding obstacles in complicated LN microenvironment.

In the specialized tissue environment of the LN paracortex, transient anchorage to restricted substrate is likely advantageous for efficient migration. Instant LFA-1 binding to ICAM-1 may serve as traction that enables T cells to achieve high velocity. Microgeometry formed by DCs and FRCs is not uniform but uneven and dynamic. In particular, the dendrites of DCs with rapid protrusions and retractions are not a stable, flat scaffold. Distribution of chemokines, ATX/LPA, and ICAM-1 are probably uneven as well. Therefore, dynamically and coordinately changing subcellular structures in T cells would be the key to adapt to microgeometry for rapid movement. Taking these into consideration, it is assumed that T cells receive intermittent migratory cues from the microenvironment, adhere to scattered anchorage spots by small cell parts, and in non-adherent sites push the cell body forward by membrane dynamics, deformation, and confinement effect (Figure 2D).

## Concluding Remarks

We especially propose that, in a “semi-confined” environment of LN, T cells dynamically switch between two motility modes, namely, anchorage-dependent motility mediated by LFA-1–ICAM-1 and anchorage-independent amoeboid movement. Unique microenvironment composed of FRCs, DCs, and lymphocytes, as well as characteristic migration modality of T cells cooperatively generate high-speed interstitial movement in the LN paracortex. However, the nanoscale view of multiple cell–cell interactions and extracellular/intracellular molecular dynamics during high-speed movement in such a complicated tissue configuration are still largely unclear. To further understand these issues, technological innovation in live imaging with much higher spatial and temporal resolutions is needed.

## Ethic Statement

All animal procedures in this study were approved by the committees on animal research at Niigata University and Kansai Medical University.

## Author Contributions

T. Katakai wrote the manuscript and designed the figures. T. Kinashi reviewed the manuscript.

## Conflict of Interest Statement

The authors declare that the research was conducted in the absence of any commercial or financial relationships that could be construed as a potential conflict of interest.

## Abbreviations

2D, two dimensional; 3D, three dimensional; ATX, autotaxin; DC, dendritic cell; FRC, fibroblastic reticular cell; INTM, intranodal T cell migration; LN, lymph node; LPA, lysophosphatidic acid.
